# E3 ubiquitin ligases LNX1 and LNX2 are major regulators of the presynaptic glycine transporter GlyT2

**DOI:** 10.1038/s41598-019-51301-x

**Published:** 2019-10-18

**Authors:** A. de la Rocha-Muñoz, E. Núñez, E. Arribas-González, B. López-Corcuera, C. Aragón, J. de Juan-Sanz

**Affiliations:** 1 Centro de Biología Molecular “Severo Ochoa”, Universidad Autónoma de Madrid, Consejo Superior de Investigaciones Científicas, 28049 Madrid, Spain; 2 IdiPAZ, Hospital Universitario La Paz, Madrid, Spain; 3 Instituto Cajal, Consejo Superior de Investigaciones Científicas, 28002 Madrid, Spain; 4 Sorbonne Université and Institut du Cerveau et de la Moelle Epinière (ICM) - Hôpital Pitié-Salpêtrière, Inserm, CNRS, Paris, France

**Keywords:** Cell biology, Cellular neuroscience, Transporters in the nervous system

## Abstract

The neuronal glycine transporter GlyT2 is an essential regulator of glycinergic neurotransmission that recaptures glycine in presynaptic terminals to facilitate transmitter packaging in synaptic vesicles. Alterations in GlyT2 expression or activity result in lower cytosolic glycine levels, emptying glycinergic synaptic vesicles and impairing neurotransmission. Lack of glycinergic neurotransmission caused by GlyT2 loss-of-function mutations results in Hyperekplexia, a rare neurological disease characterized by generalized stiffness and motor alterations that may cause sudden infant death. Although the importance of GlyT2 in pathology is known, how this transporter is regulated at the molecular level is poorly understood, limiting current therapeutic strategies. Guided by an unbiased screening, we discovered that E3 ubiquitin ligase Ligand of Numb proteins X1/2 (LNX1/2) modulate the ubiquitination status of GlyT2. The N-terminal RING-finger domain of LNX1/2 ubiquitinates a cytoplasmic C-terminal lysine cluster in GlyT2 (K751, K773, K787 and K791), and this process regulates the expression levels and transport activity of GlyT2. The genetic deletion of endogenous LNX2 in spinal cord primary neurons causes an increase in GlyT2 expression and we find that LNX2 is required for PKC-mediated control of GlyT2 transport. This work identifies, to our knowledge, the first E3 ubiquitin-ligases acting on GlyT2, revealing a novel molecular mechanism that controls presynaptic glycine availability. Providing a better understanding of the molecular regulation of GlyT2 may help future investigations into the molecular basis of human disease states caused by dysfunctional glycinergic neurotransmission, such as hyperekplexia and chronic pain.

## Introduction

Glycine acts as an inhibitory neurotransmitter in the central nervous system (CNS), playing a fundamental role in neuronal circuits of the central auditory pathway, receptive fields in the retina and spinal cord sensitive pathways. Glycinergic neurotransmission strength is controlled presynaptically by the activity of a surface glycine transporter, GlyT2, which recaptures glycine back to the presynaptic terminal to refill synaptic vesicles. Alterations in GlyT2 expression or activity result in the emptying of synaptic vesicles, which vastly weakens glycinergic neurotransmission^1–3^. In humans, this dysfunction is the main presynaptic cause of Hyperekplexia^4–6^ but may also be involved in the pathology of chronic pain^7^ and deficits in auditory processing^8^. Although the importance of GlyT2-mediated glycine transport in pathology is known^2,6,9^ and some regulatory mechanisms of this neuronal transporter have been described, a deeper understanding the molecular regulation of GlyT2 would provide insight into the molecular and cellular basis of glycinergic neurotransmission and potentially lead to identifying new therapeutic targets for Hyperekplexia or chronic pain. Previous studies on GlyT2 regulatory mechanisms revealed that GlyT2 activity is regulated by PKC activation^10,11^, P2Y and P2X purinergic receptors^12,13^ and direct interaction with several proteins^14–18^ including Na^+^/K^+^-ATPase^19^ and PMCAs^20^.

In addition, we previously described that GlyT2 trafficking and surface expression are regulated by ubiquitination^11,21^, a process in which the small protein ubiquitin is covalently attached to a cytoplasmic lysine residue of a target protein. Protein ubiquitination is a versatile regulatory post-translational modification that controls intracellular signaling events essential for neuronal function and synapse integrity, including trafficking and turnover of presynaptic proteins^22–24^. The enzymatic cascade catalyzing ubiquitination of any substrate comprises the sequential activity of the E1 ubiquitin-activating enzyme, E2 ubiquitin-conjugating enzyme and E3 ubiquitin-ligase. E3s are essential for the reaction, as they present a dual role as molecular matchmakers and catalysts to provide efficiency and specificity to the reaction^25–27^. However, despite the importance of ubiquitination in modulating GlyT2-mediated recapture of glycine, the molecular identity of E3 ligase controlling this process remains unknown.

**Figure 1:**
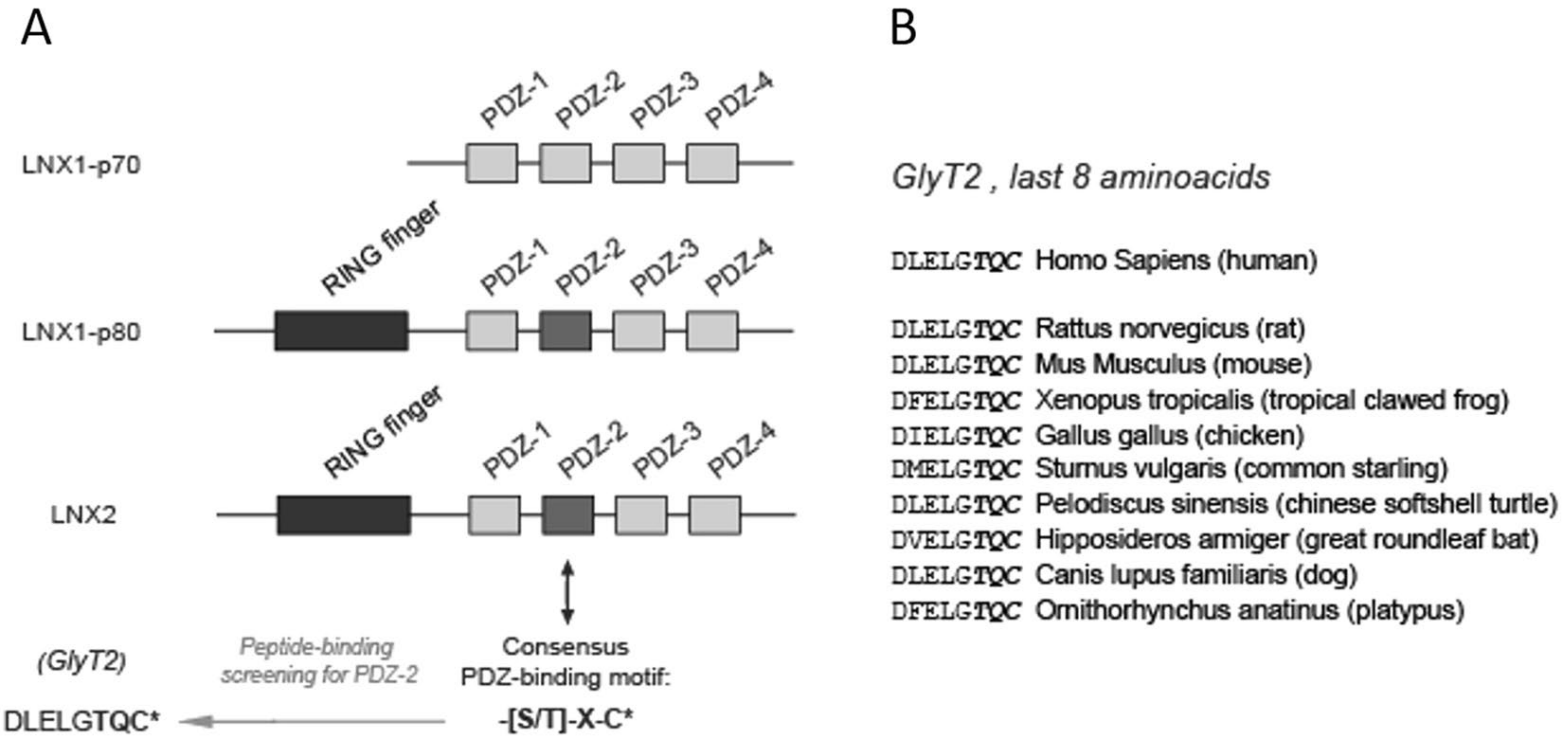
Schematic diagram of the modular domain structure of LNX1/2 proteins and sequence alignment of GlyT2 C-terminal region from different species. (**A**) Graphic scheme showing the two main isoforms of LNX1, p70 (short isoform) and p80 (long isoform with RING domain) and LNX2. A previous unbiased screening effort by Guo *et al*.^35^ identified the last 8 amino acids of GlyT2 (DLELGTQC) as a possible interacting peptide for the PDZ II domain of LNX, which recognizes the consensus sequence –[S/T]-X-C*, where * shows the end of the protein. (**B**) Multiple sequence alignment of rat GlyT2 C-terminal region 791–799 from different species. Identical conserved PDZ binding motif (PBM) from different species are shown in bold.

The LNX (Ligand of NUMB Protein-X) protein family is a family of E3 ubiquitin ligases characterized by the presence of a RING domain and one to four PDZ domains^28^. LNX1 and LNX2, two of the five members of the family, are expressed in neurons and other cell types in the nervous system^28^ and present high structural homology with one RING and four consecutive PDZ domains (Fig. 1A). These PDZ domains promote the interaction with many neuronal substrates such as NUMB^28^, c-Src^29^ or PKCα^30^, as well as the presynaptic active zone proteins CAST^31^, ERC1, ERC2 and LIPRIN-αs^32^, which has led to suggest that LNX1/2 may modulate synapse maturation and neurotransmission^33,34^. To better understand the modulatory roles of LNX1/2 in neurons and other cell types, previous efforts in the field took advantage of unbiased proteomic screening approaches to identify potential interactors of each of the PDZ domains^32,35–37^. The second PDZ domain of LNX1/2 (PDZ2) is a class I PDZ domain that binds C-terminal motifs with the sequence S/T-X-C^35^, a compatible sequence with the highly conserved PDZ binding motif existing in the C-terminus of GlyT2 (sequence TQC, see Fig. 1B). A previous proteomic study unbiasedly identified the possibility of an interaction between LNX1 and GlyT2^35^, although no validation or functional studies were pursued after the initial proteomic identification. Given the importance of GlyT2 in the control of inhibitory glycinergic neurotransmission and that ubiquitination is an essential post-translational modification that regulates its function and expression, we decided to explore whether LNX1 and the homologous LNX2 are presynaptic E3-ligases that control GlyT2 activity.

In this work, we demonstrate that GlyT2 interacts with and it is ubiquitinated by LNX1 and LNX2 (LNX1/2). LNX1/2 overexpression reduces the expression of GlyT2, impairing glycine transport. Mutations in the C-terminal lysine cluster of GlyT2 prevent both ubiquitination and effects on GlyT2 activity induced by wild type LNX1/2, indicating that the last 4 lysines are necessary for the modulatory function of LNX1/2 onto GlyT2. Contrarily to the effects observed in overexpression experiments, genetic ablation of LNX2 by shRNA in primary spinal cord neurons caused an increase in the expression of GlyT2, indicating the existence of a constitutive regulation of the transporter by LNX2 activity in glycinergic neurons. Previous studies identified that PKC activation resulted in increased GlyT2 ubiquitination and decreased expression^10,11,21^. Here we show that ablation of LNX2 in spinal cord neurons abolished the effect of PKC on the expression of GlyT2, suggesting that PKC is a key controller of LNX1/2 activity and its effect on GlyT2 expression. Taken together, these findings indicate that LNX1/2 can modulate presynaptic glycine recapture by regulating GlyT2 ubiquitination levels and expression, suggesting that these proteins may play a role in controlling inhibitory glycinergic neurotransmission strength in the CNS.

## Results

### LNX1 p80 and p70 isoforms interact with GlyT2

**Figure 2:**
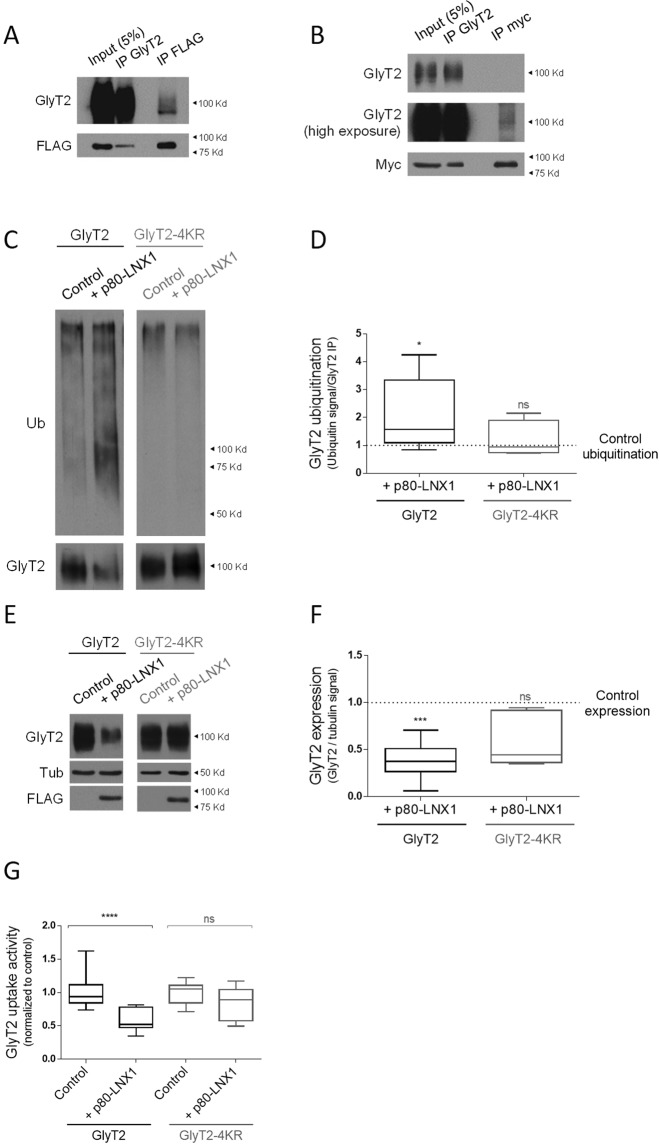
The interaction of LNX1 with GlyT2 controls the functional expression of the transporter. (**A,B**) COS7 cells lysates expressing GlyT2 and FLAG-p80LNX1 (**A**) or Myc-p70LNX1 (**B**) were subjected to immunoprecipitation against GlyT2 and FLAG or Myc, showing that GlyT2 coimmunoprecipitates both LNX1-p70 (**B**, middle lane) and LNX1-p80 (**A**, middle lane). Correspondingly, LNX1-p70 and LNX1-p80 coimmunoprecipitate GlyT2 (**A,B**, right lane). A higher exposure is shown in the case of p70 to observe GlyT2 in Myc-LNX1-p70 immunoprecipitates. C) COS7 cells were transiently transfected with GlyT2 or GlyT2-4KR, HA-tagged ubiquitin and with or without FLAG-LNX1-p80. GlyT2 was immunoprecipitated and ubiquitination of the transporter was assayed by immunoblotting against HA. Blots were probed against GlyT2 to normalize ubiquitination signal against the amount of GlyT2 immunoprecipitated in each case to correct for GlyT2 protein expression. (**D**) Quantification of GlyT2 ubiquitination normalized to the control (no transfection of LNX1). *p = 0.011, n.s., not significantly different, using Kruskall-Wallis with Dunn’s post hoc test. Number of experiments in each case: n(GlyT2 control) = 13, n(GlyT2-p80) = 13, n(GlyT2-4KR control) = 4, n(GlyT2-4KR-p80) = 4. Blots used in this figure are obtained from different gels, and cropped results are presented for ease of visualization. (**E**) COS7 cells were transiently transfected with GlyT2 or GlyT2-4KR in combination with pcDNA3 or LNX1-p80 and GlyT2 expression was measured by immunoblotting. Quantification of the effect of LNX1-p80 on GlyT2 and GlyT2-4KR expression was normalized against tubulin. (**F**) Quantification is shown normalized to the corrected signal in the control in each case with no LNX1-p80 co-transfection (indicated by dashed line). ***p = 1 · 10^−4^, n.s., not significantly different, using Kruskall-Wallis with Dunn’s post hoc test. n(GlyT2 control) = 10, n(GlyT2-p80) = 10, n(GlyT2-4KR) = 5, n(GlyT2-4KR-p80) = 5. Note that while LNX1-p80 induces a reduction of GlyT2 expression, GlyT2-4KR is highly protected in the same conditions. Effect of LNX1-p80 in wild type GlyT2 expression is shown here to ease comparison of the effects in GlyT2-4KR. FLAG immunobloting was used to confirm transfection of LNX1. (**G**) COS7 cells were transiently transfected as in (**C**) and glycine transport rates were measured using [^3^H]-glycine transport assays. Glycine transport shown is normalized against control conditions in each case. ****p < 1 · 10^−4^, n.s., not significantly different, using Kruskall-Wallis with Dunn’s post hoc test. Number of experiments in each case: n(GlyT2 control) = 43, n(GlyT2-p80) = 27, n(GlyT2-4KR control) = 16, n(GlyT2-4KR-p80) = 16.

To explore whether LNX1/2 could be E3 ligases for GlyT2, we began by exploring the physical and functional relationship between LNX1 and GlyT2. First, to study whether LNX1 and GlyT2 interact in heterologous cells, we transfected COS7 cells with GlyT2 and FLAG-tagged LNX1-p80 isoform (LNX1-p80, the longest LNX1 isoform; see Fig. 1A), and used coimmunoprecipitation assays to detect protein interaction. Immunoprecipitation of GlyT2 resulted in coimmunoprecipitation of FLAG-tagged LNX1-p80 (Fig. 2A, second lane), and correspondingly, immunoprecipitation of FLAG-tagged LNX1-p80 coimmunoprecipitated GlyT2 (Fig. 2A, third lane). We also explored whether LNX1-p70, a shorter isoform with no E3 ligase activity (Fig. 1A), interacts with GlyT2, as this isoform also contains the PDZ2 domain that was predicted to bind the transporter. Using the same approach, we observed that immunoprecipitation of GlyT2 resulted in coimmunoprecipitation of Myc-tagged LNX1-p70 (Fig. 2B, second lane), and correspondingly, immunoprecipitation of Myc-tagged LNX1-p70coimmunoprecipitated GlyT2 (Fig. 2B, third lane; see high exposure). These data indicate that the previously identified interaction between the PDZ2 domain of LNX1 and a purified peptide with the sequence of the last 8 amino acids of GlyT2^35^ does in fact occur between full-length GlyT2 and LNX1 in a cellular environment.

### Ubiquitin ligase activity of LNX1 regulates GlyT2 expression and function

We next hypothesized that the observed LNX1-GlyT2 interaction may occur to control the ubiquitination state of GlyT2. We assayed whether LNX1 ubiquitinates GlyT2 in cells by co-transfecting HA-tagged ubiquitin and GlyT2 with or without overexpressing wild type LNX1-p80 in COS7 cells. We immunoprecipitated GlyT2 from cell lysates and measured its ubiquitination levels by immunoblotting with an anti-HA antibody, normalizing HA immunodetection against the immunoprecipitated amount of GlyT2 in each case to control for variability on GlyT2 expression. Compared to the control (Fig. 2C lane 1), an increased ubiquitination signal was found when LNX1-p80 was overexpressed (Fig. 2C, lane 2) showing that increased levels of LNX1 indeed promote GlyT2 ubiquitination. These experiments also show that GlyT2 expression levels are drastically reduced when LNX1-p80 is overexpressed (Fig. 2C lower blot; discussed in next paragraph). Quantification of these data indicated that overexpression of LNX1 significantly increased GlyT2 ubiquitination levels by ~2 fold (Fig. 2D, n = 13, *p = 0.011GlyT2). Next, we explored whether this process requires a cytoplasmic C-terminal lysine cluster in GlyT2 that has been previously demonstrated to control its ubiquitination and expression^21^. Using the same approach, we expressed a mutant in which this cluster of lysines have been mutated to arginines to impair their ubiquitination (GlyT2-4KR) with or without LNX1-p80 and we found no significant differences in ubiquitination levels of GlyT2-4KR when LNX1-p80 was overexpressed (Fig. 2C, lane 4; n = 5, n.s., not significantly different). These results indicate that LNX1-p80 ubiquitinates the C-terminal lysine cluster of GlyT2.

Our ubiquitination experiments did not find single bands corresponding to monoubiquitinated GlyT2 forms but showed a typical smear pattern that likely corresponds to polyubiquitinated forms^38,39^. Whereas monoubiquitination in general may regulate location and activity of diverse cellular proteins^40^, polyubiquitination is the type of ubiquitin modification that can target proteins for degradation^41^. We noticed that in our ubiquitination experiments overexpression of LNX1-p80 induced a significant reduction in GlyT2 expression levels, suggesting that ubiquitination of GlyT2 by LNX1 regulates the expression of the transporter, similarly to what it does with other proteins^35,42–46^. Results from Fig. 2E,F showed that co-expression of LNX1-p80 and GlyT2 resulted in a significant reduction of ~60% in the levels of GlyT2 (n = 10, ***p = 1 · 10^−4^) but no variation in the expression of GlyT2-4KR was detected. These results indicate that LNX1 can only control GlyT2 expression if the cytoplasmic C-terminal lysine cluster in GlyT2 remains intact, confirming that LNX1 promotes ubiquitination of those particular residues to control GlyT2 expression. To understand the functional relevance of LNX1-mediated control of GlyT2 expression, we next explored to what extent LNX1 expression impacts glycine recapture using [^3^H]-glycine uptake assays in COS7 cells. Glycine transport by GlyT2 is essential for maintaining diverse aspects of motor function and impairment of its activity causes important pathologies in humans, from movement disorders to sensory dysfunctions^9^. We observed that co-expression of wild-type LNX1-p80 with GlyT2 resulted in a significant reduction of glycine transport of ~40% (Fig. 2G, n(GlyT2 control) = 43, n(GlyT2- LNX1-p80) = 27; ****p < 1 · 10^−4^). Contrarily to wild type GlyT2, transport activity of GlyT2-4KR remained unaffected with LNX1-p80 overexpression (Fig. 2G, right, n(GlyT2-4KR control) = 16, n (GlyT2-4KR-LNX1-p80) = 16), indicating that LNX1-p80 alters the transport activity of GlyT2 by reducing the expression of the transporter. Taken together, these results indicate that LNX1-p80 induces ubiquitination of the C-terminal lysine cluster of GlyT2 and this process regulates the expression levels and transport activity of GlyT2.

### LNX2 also interacts with GlyT2 to control its ubiquitination and expression

**Figure 3:**
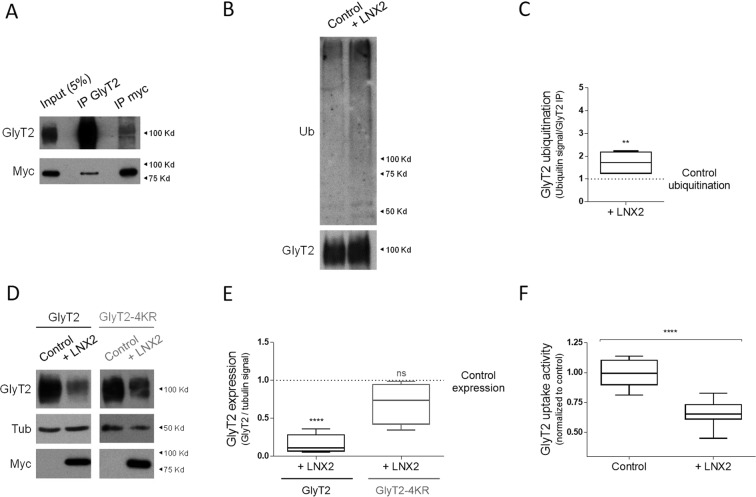
Identification and characterization of LNX2 as interacting partner of GlyT2. (**A**) COS7 cells expressing GlyT2 and Myc-LNX2 were lysed and subjected to immunoprecipitation against GlyT2 and Myc. Immunoprecipitates were analyzed by immunoblotting for indicated proteins, showing that GlyT2 coimmunoprecipitates LNX2 (middle lane). Correspondingly, LNX2 coimmunoprecipitate GlyT2 (right lane). (**B**) COS7 cells were transiently transfected with GlyT2 and with or without Myc-LNX2. GlyT2 was immunoprecipitated and ubiquitination of the transporter was assayed by immunoblotting with anti-ubiquitin antibody. Blots were probed against GlyT2 to normalize ubiquitination signal against the amount of GlyT2 immunoprecipitated in each case to correct for GlyT2 protein expression. (**C**) Quantification of GlyT2 ubiquitination normalized to the control (no transfection of Myc-LNX2). **p = 0.022, using Kolmogorov-Smirnov test. n(control) = 6, n(LNX2) = 6. (**D**) COS7 cells were transiently transfected with GlyT2 or GlyT2-4KR in combination with pcDNA3 or Myc-LNX2 and GlyT2 expression was measured by immunoblotting. Quantification of the effect of LNX2 on GlyT2 and GlyT2-4KR expression was normalized against tubulin. (**E**) Quantification is shown normalized to the corrected signal in the control in each case with no LNX2 co-transfection (indicated by dashed line). ****p < 1 · 10^−4^, n.s., not significantly different, using Kruskall-Wallis with Dunn’s post hoc test. Number of experiments in each case: n(GlyT2 control) = 8, n(GlyT2-LNX2) = 8, n(GlyT2-4KR) = 4, n(GlyT2-4KR-LNX2) = 4. Note the higher reduction of GlyT2 expression by LNX2 respect to that induced in GlyT2-4KR mutant. Myc immunobloting was used to confirm transfection of LNX2. (**F**) COS7 cells were transiently transfected with GlyT2 in combination with pcDNA3 or Myc-LNX2 and glycine transport rates were measured using [^3^H]-Glycine transport assays as described inexperimental procedures. Glycine transport shown is normalized against control conditions. ****p < 1 · 10^−4^, n.s., not significantly different, using Mann-Whitney U test. n(control) = 27, n(LNX2) = 27.

LNX2 presents high structural homology to the largest isoform of LNX1, LNX1-p80 (Fig. 1A). Given that many interactors of the PDZ2 domains of LNX1 and LNX2 are shared between both proteins^28,30,33^, we next explored whether LNX2 could also act as a regulator of GlyT2 activity. We followed a similar approach to the one followed for LNX1. First, using coimmunoprecipitation assays performed in COS7 cells expressing GlyT2 and Myc-tagged LNX2 we confirmed that both proteins interact, as the immunoprecipitation of GlyT2 resulted in coimmunoprecipitation of Myc-tagged LNX2 (Fig. 3A, lane 2), and correspondingly, immunoprecipitation of Myc-tagged LNX2 coimmunoprecipitated GlyT2 (Fig. 3A, lane 3). Next, we performed ubiquitination assays as before. GlyT2 was immunoprecipitated from cell lysates and its level of ubiquitination was analyzed by immunoblotting with an anti-ubiquitin antibody (Fig. 3B). Compared to the control (Fig. 3B lane 1) an increased ubiquitination signal was measured 24 h after transfection when LNX2 was overexpressed (Fig. 3B lane 2) and quantification of these data indicated that LNX2 significantly increased GlyT2 ubiquitination levels by ~1.5 fold (Fig. 3C, n = 6; **p = 0.022). Consistent with the idea that LNX2 functions as E3 ubiquitin ligase^26^, we observed that co-expression of Myc-LNX2 with GlyT2 resulted in a significant reduction of ~60% in the level of GlyT2 and a negligible variation in GlyT2-4KR expression (Fig. 3D,E n(GlyT2 control) = 8, n(GlyT2 + LNX2) = 8, n(GlyT2-4KR) = 4, n (GlyT2-4KR + LNX2) = 4; ****p < 1 · 10^−4^). However, we also observed that despite both LNX1 and LNX2 ubiquitinate GlyT2 to control its expression, LNX2 effects appeared to be slower and degradation of the transporter was only seen after 48 h of overexpression of the ubiquitin ligase (Fig. 3D,E shows expression at 48 h; for no effect at 24 h see blot for GlyT2 in Fig. 3B). Lastly, we confirmed that LNX2 activity can impact glycine recapture using [^3^H]-glycine transport assays. GlyT2 transport activity showed a decrease of ~40% when LNX2 was overexpressed (Fig. 3F) n(control) = 27, n(LNX2) = 27. ****p < 1 · 10^−4^. Thus, these results confirm that the E3 ubiquitin ligase LNX2 interacts with GlyT2 to modulate its expression and function through ubiquitination.

### Ablating LNX2 in neurons increases the expression of endogenous GlyT2

**Figure 4:**
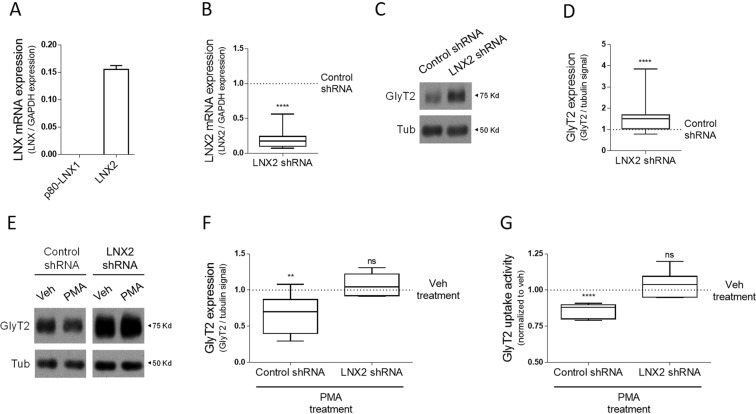
Silencing of LNX2 in neurons causes upregulation of endogenous GlyT2 and abolish the PKC-mediated regulation of GlyT2. (**A**) Relative mRNA levels of LNX1-p80 and LNX2 were determined by qPCR using Glyceraldehyde-3-phosphate dehydrogenase (GAPDH) as housekeeping gene (arbitrary units). n(kidney) = 3, n(primary brainstem and spinal cord neuron cultures) = 3. (**B**) Neuronal cultures were infected with LNX2 shRNA and scrambled shRNA as control. After 12 DIV total RNA was extracted from cells and quantification of LNX2 mRNA was determined by qPCR. LNX2 mRNA in these conditions decreased by 81.4 ± 11,8%. ****p < 1 · 10^−4^, n.s., not significantly different, using Kolmogorov-Smirnov test. n(control shRNA) = 14, n(LNX2 shRNA) = 14. (**C**) Cultured neurons were infected as in (**B**) and western blot analysis were performed using anti-GlyT2 antibodies. Tubulin was used as a loading control. (**D**) Quantification is shown normalized to the corrected signal in the control (scrambled shRNA) indicated by a dashed line. ****p < 1 · 10^−4^, n.s., not significantly different, using Kolmogorov-Smirnov test. n(control shRNA) = 16, n(LNX2 shRNA) = 16. (**E**) Neuronal cultures infected with LNX2 shRNA or scrambled shRNA were treated with or without (vehicle) 1 µM PMA during 2 h and were subjected to western blot analysis using anti-GlyT2 antibodies. Tubulin was used as protein loading control. Note that ablation of LNX2 impairs the effect of PMA on GlyT2 expression. (**F**) Quantification is shown normalized to the corrected signal in the control (vehicle) indicated by dashed line. **p = 0,005, n.s., not significantly different, using Two-way Anova with Sidak’s post hoc test. Number of experiments in each case: n(control shRNA + Veh) = 7, n(control shRNA + PMA) = 7, n(LNX2 shRNA + Veh) = 6, n(LNX2 shRNA + PMA) = 6. (**G**) In neuronal cultures infected and PMA treated as in E glycine transport rates were measured using [^3^H]-Glycine transport assays. Glycine transport shown is normalized against control conditions in each case. ****p < 1 · 10^−4^, n.s., not significantly different, using Two-way Anova with Sidak’s post hoc test. Number of experiments in each case: n(control shRNA + Veh) = 6, n(control shRNA + PMA) = 6, n(LNX2 shRNA + Veh) = 5, n(LNX2 shRNA + PMA) = 5.

We next decided to explore whether neuronal GlyT2 is modulated by endogenous LNX proteins. We first measured the expression of LNX1 and LNX2 using qPCR in primary cultures of brainstem and spinal cord, in which GlyT2 is strongly expressed. Relative mRNA levels of LNX proteins were quantified and normalized using glyceraldehyde-3-Phosphate Dehydrogenase (GAPDH, see experimental procedures). Although the mRNA of LNX2 was easily detectable, we could not detect the mRNA of LNX1-p80 in DIV12 primary neurons (Fig. 4A). LNX1 is strongly expressed in neuronal stem cells^47^ but it expression is known to decay in differentiated neurons^28,46^. To confirm that the lack of detection of LNX1-p80 in our cultures was not due to a technical issue, we verified that our probes could easily detect the mRNA of p80-LNX1 in renal tissue, as previously published^28^ (not shown). Because of the apparent scarce or undetectable presence of the catalytically active isoform LNX1-p80 in our system we decided to only explore the role of endogenous LNX2 in controlling GlyT2. To do this, we ablated the expression of LNX2 using a viral knockdown approach in primary neurons that reduced LNX2 mRNA by 80% after 11 days of infection when compared to neurons infected with a scrambled shRNA (Fig. 4B). Notably, in these conditions ablating the function of LNX2 resulted in a significant increase of endogenous GlyT2 expression (Fig. 4C,D; n = 6; ****p < 1 · 10^−4^). This supports our initial experiments in heterologous cells as in these, overexpression of LNX2 causes a decrease in GlyT2 expression and correspondingly in neurons LNX2 ablation results in an increase of the transporter.

### LNX2 is required for PKC-mediated regulation of GlyT2 activity

Previous work showed that activation of PKC in glycinergic neurons results in an increased ubiquitination of GlyT2 and reduction of its expression and transport activity, suggesting that this may be a control mechanism of glycinergic neurotransmission strength^10,11,21^. PKC is a well-known regulator of neurotransmitter release in many synapse types in the CNS and its activation by phorbol esters has been classically used to dissect its essential role in the process of neurotransmitter release^48–50^. We reasoned that, since LNX2 appears to be a major controller of GlyT2 ubiquitination status, the effect of PKC activation in controlling GlyT2 function may require LNX2. To explore this, we ablated LNX2 expression in glycinergic neurons using shRNA as before and treated these cultures with PMA (Phorbol 12-Myristate 13-Acetate), a known activator of PKC. While PKC activation in control neurons resulted in a reduction of GlyT2 levels by ~30% as expected (Fig. 4E, left), ablating LNX2 significantly blocked the effect of PMA (Fig. 4E, right; Fig. 4F shows quantification; n = 7 **p = 0,005). To confirm the impact of this modulation in the glycine transport activity of GlyT2, we measured [^3^H]-glycine transport uptake in primary neurons from brainstem and spinal cord in the same conditions. In agreement with expression experiments, transport by GlyT2 was significantly reduced (Fig. 4G; n = 6; ****p < 1 · 10^−4^) but no variation was observed in LNX2 depleted neurons. Taken together these experiments show that LNX2 is essential for the control of GlyT2 by PKC in glycinergic neurons, providing the missing molecular link between PKC activation and acceleration of GlyT2 ubiquitination.

## Discussion

This study provides compelling novel evidence demonstrating that the RING-finger E3 ubiquitin ligases LNX1 and LNX2 are functional regulators of the neuronal glycine transporter GlyT2. Our data from immunoprecipitation, ubiquitination and glycine transport assays reveal that LNX1 and LNX2 interact with GlyT2 and ubiquitinate a C-terminal cluster of lysines of this transporter to control its expression and activity. Using virally-delivered shRNAs we confirmed that endogenous LNX2 controls the expression of GlyT2 in glycinergic neurons, both constitutively and during PKC-mediated activation of GlyT2. Our work presents, to our knowledge, the first identification of an E3 ligase controlling GlyT2 ubiquitination, expression levels and activity.

Presynaptic GlyT2 activity is essential for sustaining glycinergic neurotransmission strength^1–3^ and its dysregulation is strongly associated with neurological disease^5,51^. Our identification of LNX1 and LNX2 as major controllers of GlyT2 suggests that these molecules may have a pathophysiological role in controlling glycinergic transmission by modulating presynaptic glycine recapture. LNX1/2 also interact with several other presynaptic proteins including CAST^31^, NUMB^42,46^, c-Src^29^, PKCα^30^, ERC1, ERC2 and LIPRIN-αs^32^. The interactional promiscuity of the LNX family suggests that LNX proteins may modulate presynaptic biology by coordinately tuning the expression of a diverse set of molecules. LNX1/2 present four PDZ domains with different recognition sequences^52–54^, which supports the idea that they can recognize and ubiquitinate many presynaptic interactors^29–32^. Although speculative, a central role of LNX1/2 in coordinating presynaptic ubiquitination could act as a mechanism for the parallel adjustment of the function of several molecules controlling presynaptic biology. Such a mechanism would facilitate the choreographed modulation of the presynaptic molecular landscape, which could constitute a molecular mechanism to control on demand the strength of synaptic transmission.

On the other hand, given that LNX1/2 are multi-PDZ proteins, they may also act as molecular scaffolds to bring together their interacting partners to the same specific subcellular location^30,52^. LNX1/2 could coordinate the presynaptic formation of multimolecular complexes of GlyT2 and other interacting partners^28,31^, facilitating the subcellular positioning and function of these proteins. GlyT2 has been previously shown to interact with several presynaptic proteins, including syntaxin1^16^, Plasma Membrane Calcium ATPases PMCA2 and PMCA3^20^ and sodium/potassium ATPase subunits α3 (α3NKA) and β2 (β2NKA)^19^. It is possible that LNX1/2 may act as scaffold to facilitate the interaction of GlyT2 with these proteins, an idea supported by a recent proteomic study that found PMCA2 and β2NKA, known interactors of GlyT2^19,20^, as interacting partners of LNX1^32^.

A surprising fact in the LNX family is that, despite that LNXs mRNAs are easily detected^28,46,55^, LNX proteins are present at low levels in many adult tissues^29,43,55^. The low protein levels of LNX proteins may allow to dynamically regulate their activity by simply generating novel protein through the existing mRNA. This putative mechanism would not be unique to LNX proteins, and in fact it has been already described that increased translation of existing transcripts can help to quickly synthesize newly required proteins to control short-term temporal adaptation mechanisms^56^. Even more, E3 ligase-substrate interactions are in many cases constitutive, and, in such cases, regulation is thought to occur at the level of E3 transcription/translation or degradation^57^. Such mechanistic control of LNXs proteins presynaptically could be particularly relevant for neuronal function, as modulation of presynaptic ubiquitination has been shown to acutely regulate neurotransmitter release in mammalian neurons^58^.

Our shRNA experiments in glycinergic neurons reveal that LNX2 constitutively controls GlyT2 expression. However, it is essential to also understand in which conditions a glycinergic synapse may modulate this existing pathway to correspondingly control presynaptic glycine recapture by GlyT2. LNX1 domains PDZ2 and PDZ4 interact with Ca^2+^-dependent protein kinase C isoform α^30^, a known regulator of presynaptic short-term plasticity^48^. Our data indicates that PKC activation accelerates ubiquitination of GlyT2 by LNX2, suggesting that this may be an additional mechanism that contributes to the effects of PKC activation in neurotransmitter release in a glycinergic synapse^59^. Given that other neurotransmitter transporters are also regulated by PKC^60–64^ and ubiquitination^61,65,66^, it is possible that LNX2 may act as a controller of the activity of these transporters during PKC activation. Future work, however, will be required to dissect this possibility.

Ubiquitination is a major pathway that controls turnover of neuronal membrane proteins^24^. Although ubiquitination is now considered a major regulatory pathway that controls GlyT2 function^9,11,21^, the molecular identity of the enzymes catalyzing GlyT2 ubiquitination has remained elusive. To our knowledge, the work presented here identifies the first E3-ubiquitin ligases acting on GlyT2. We discovered that the E3 ubiquitin ligases LNX1 and LNX2 modulate the ubiquitination status of GlyT2, ubiquitinating its cytoplasmic C-terminal lysine cluster in a process that regulates the expression levels and transport activity of the transporter in neurons. Correct maintenance of glycine transport by GlyT2 is essential for human physiology, as GlyT2 is an essential regulator of glycinergic neurotransmission strength. Our identification of LNX1 and LNX2 as novel regulators of GlyT2 may have pathophysiological relevance on the biology of glycinergic neurotransmission and might help frame future investigations into the molecular basis of human disease states that are consequence of dysfunctional glycinergic neurotransmission, such as hyperekplexia and chronic pain.

## Methods

### Materials

Male Wistar rats were bred under standard conditions at the Centro de Biología Molecular Severo Ochoa (CBMSO) in accordance with procedures approved in the Directive 2010/63/EU of the European Union with approval of the Research Ethics Committee of the Universidad Autónoma de Madrid (Comité de Ética de la Investigación UAM, CEI-UAM). Antibodies against GlyT2 N-terminus were generated in house (rabbit and rat^67,68^) while the other primary antibodies used were: anti-c-Myc (Myc-Tag 9B11 Cell Signaling Tech.), anti-FLAG M2 (Sigma-Aldrich, F3165), anti-HA (Sigma-Aldrich, clon 12CA5), anti-ubiquitin (P4D1, Santa Cruz), anti-α-tubulin (Sigma-Aldrich, T6074). All chemicals used were from Sigma Aldrich unless otherwise noticed. Neurobasal medium and B27 supplement were purchased from Invitrogen.

### Plasmids, small hairping RNAs (shRNAs) and lentiviral particles generation

FLAG-tagged LNX1-p80 was a generous gift from Prof. Jane McGlade (University of Toronto), pClneoMyc mouse LNX1-p70 and pClneoMyc mouse LNX2 were a gift from Yutaka Hata (Addgene plasmids #37009 and #37010, respectively) and pRK5-HA-Ubiquitin-WT was a gift from Ted Dawson (Addgene plasmid #17608). Generation of GlyT2-4KR was described in previous work^16,21^. GlyT2 constructs are based on the longest rattus novergicus isoform of 799 aminoacids (Slc6a5-201, Ensembl transcript ID ENSRNOT00000041950.4). The shRNA sequence against LNX2 was designed by the The Genetic Perturbation Platform at the Broad Institute of Harvard and MIT (Massachusetts, USA) with the identification number TRCN0000040715. Since LNX1 and LNX2 present very short half-lives (see Supp. Fig. 1) and only last for 1–3 days after transfection, but the shRNA needs to be expressed for 11 days for efficient knock-down, it resulted technically challenging to match and re-express the wild-type LNX2 in the shLNX2 background to rescue the function as a control for the shRNA specificity. shRNA lentiviral particles were generated by transfection of HEK293T with the pLKO vectors containing shRNA, the packaging plasmid psPAX2 and the envelope plasmid pMD2G. The sequence of the shRNAs were as follows:

Scramble shRNA:

5′ -CCTAAGGTTAAGTCGCCCTCGCTCGAGCGAGGGCGACTTAACCTTAGG-3′

LNX2 shRNA:

5′ -CCACTGATCAACATCGTCATT-3′

### Cell growth and protein expression

COS7 cells (American Type Culture Collection) were grown at 37 °C and 5% CO2 in Dulbecco’s modified Eagle’s medium (DMEM) supplemented with 10% fetal bovine serum. Transient expression was achieved using Turbofect Transfection Reagent (Fisher Scientific), according to the manufacturer’s protocol, and cells were then incubated for 24–48 h at 37 °C. Reproducible results were obtained with 80–90% confluent cells on 60-mm or 6-well plates, using 5 and 2 µg of total DNA, respectively. The ratio of DNA between GlyT2 and LNX1/2 variants was 1:4 to favor LNX1/2 overexpression.

### Primary cultures of brainstem and spinal cord neurons and infection

Primary cultures of brainstem and spinal cord neurons were prepared as described previously^19^. Briefly, the brainstem and spinal cord of Wistar rat fetuses were obtained at the 16th day of gestation, and the tissue was then mechanically disaggregated in HBSS (Invitrogen) containing 0.25% trypsin (Invitrogen) and 4 mg/ml DNase (Sigma). Cells were plated at a density of 500,000 cells/well in 12 well multiwell plates (Falcon), and they were incubated for 4 h in DMEM containing 10% FCS, 10 mM glucose, 10 mM sodium pyruvate, 0.5 mM glutamine, 0.05 mg/ml gentamicin, 0.01% streptomycin and 100 U/ml penicillin G. After 4 h this buffer was replaced with Neurobasal/B27 culture medium containing 0.5 mM glutamine (50:1 by volume: Invitrogen), and 2 days later cytosine arabinoside (1 µM) was added to inhibit further glial growth. Neuronal cultures were infected with lentiviruses at DIV 1. Lentivirus preparations were added directly to the culture medium and maintained for 24 h at 5% v/v. Neurons were then replaced in fresh medium and culture was continued until 12 DIV.

### Quantitative Real-Time PCR (qPCR)

Total RNA was extracted from cultured rat brainstem and spinal cord neurons following the TRI Reagent isolation protocol (Sigma-Aldrich).

cDNA was synthetized using the iScript cDNA Synthesis kit (Bio-Rad) and qPCR was performed using Fast SYBR Green Master Mix (Thermofisher Scientific) following manufacture’s recommendations. Relative gene expression levels were quantified by the 2^−∆∆Ct^ method using Glyceraldehyde-3-phosphate dehydrogenase (GAPDH) as housekeeping gene.

Primer sequences:

GAPDH:

5′ -TCCCATTCTTCCACCTTTGA-3′ 5′ -ATGTAGGCCATGAGGTCCAC-3′

LNX1:

5′ -TGGAGGCGGGCTGGTGA-3′ 5′ -TTCCTCAGGGGCAGGTCAAGA-3′

LNX2:

5′ -CCGTGTGCCAAGATGTAATG-3′ 5′ -GGATCCAGTTTCACCCTCAA-3′

### Immunoprecipitation and western blotting

COS7 cells or primary cultures of brainstem and spinal cord were lysed for 30 min at room temperature (RT) at a concentration of 1.5 mg of protein/ml in TN buffer (25 mM Tris HCl and 150 mM NaCl [pH 7.4]) containing 0.25% Nonidet P-40 (NP-40) and protease inhibitors (PIs: 0.4 mM phenylmethylsulfonyl fluoride (PMSF) and Sigma cocktail). After 15 min centrifugation in a microfuge to remove the cell debris, 4% of protein was separated to quantify total protein (T) and 5 μl of the primary antibody were added and left overnight at 4 °C using the following antibodies for immunoprecipitation: rat anti-GlyT2, anti-c-Myc or anti-FLAG M2. A negative control was also run in parallel in which no antibody was added. Subsequently, 50 μl of 50% protein G agarose beads (PGA; ABT beads Inc.) were added and incubated for 45 min at 4 °C. The beads were collected by mild centrifugation and washed twice for 7 minutes with lysis buffer at RT. Finally, the beads were pelleted and the immunoprecipitated proteins were eluted in Laemmli buffer at 75 °C for 10 min, resolved in SDS/PAGE gels (7.5%), detected in Western blots by enhanced chemiluminescence (ECL). As previously reported, Western blot against GlyT2 obtained from COS7 cells lysates identified several bands corresponding to fully glycosylated functional transporter (100 KDa) and partially non-glycosylated intracellular forms (75 KDa) that tends to aggregate (150 KDa) due to having incomplete glycosylation^18^. In the main figures of this study we present the bands corresponding to the functional forms of GlyT2 (100 KDa) but supplementary figures show uncropped blots where all forms of the transporter can be visualized. In neurons, however, GlyT2 only appears in the form of the functional transporter.

### Ubiquitination assay

COS7 cells were transiently transfected with plasmids encoding wild-type or mutant GlyT2 proteins and with/without HA-tagged ubiquitin. 24–48 hours after transfection, cells were treated for 3 hours at 37 °C with leupeptine 100 μM. Cells were washed twice with PBS at 4 °C, harvested using a buffer containing 50 mM Tris, 150 mM NaCl and 50 mM N-ethylmaleimide with protease inhibitors PMSF and Sigma protease inhibitor cocktail (Ubiquitination Buffer, UB) and analyzed for protein quantification. Equal amounts of protein samples were centrifuged and pellets were resuspended in 90 microliters of UB. 10 microliters of 10% sodium dodecyl sulphate (SDS) were added and incubated for 10 min at 95 °C to eliminate protein interactions. Then, 34 microliters of UB + 4% Triton and 1 ml of UB + 1%Triton X100 was added for 30 min at 4 °C and centrifuged. Lysates were precleared with 40 microliters of ProteinG-agarose for 30 min at 4 °C and then were incubated overnight with anti-GlyT2, coupled to ProteinG-agarose 1 h at room temperature followed by four washes with ice-cold lysis buffer and elution in 2x Laemmli sample buffer. Cell lysates were probed by Western blot analysis with specific antibodies against GlyT2, HA or ubiquitin (P4D1, Santa Cruz). We used anti alphaTubuline (sigma T-6074) as a loading control. Primary neurons infected with LNX2 shRNA or scrambled shRNA were subjected to the same procedure described for COS7 cells.

### [^3^H]-Glycine transport assays

For assaying GlyT2 transport activity, cells were incubated in a solution with an isotopic dilution containing 2 μCi/ml [^3^H] glycine (1.6 TBq/mmol; PerkinElmer Life Sciences) in PBS, yielding a 10 μM final glycine concentration. To measure unspecific glycine accumulation (background), the transport assay was also performed in the presence of the GlyT2 antagonist ALX1393 (0.4 μM, IC50 = 50 nM). Transport was measured by subtracting the background glycine accumulation in COS7 cells or primary neurons and normalizing to the protein concentration and time of the reaction. Experiments in neurons were performed in the presence of the GlyT1 antagonist NFPS (10 μM) as these cultures present GlyT1 endogenous activity that would contaminate the measurements of GlyT2 transport activity. The reactions were terminated after 10 min by aspiration, followed by washing with HBS.

### Densitometry, data analysis and statistics

The protein bands obtained by ECL (Bio-Rad) using film exposures in the linear range were imaged using a GS-900 calibrated imaging densitometer (Bio-Rad) and quantified using Image Lab Software (Bio-Rad). All statistical analyses were performed using GraphPad Prism (GraphPad Software). Kruskal-Wallis was used to compare multiple groups, with subsequent Dunn’s post-hoc test to determine the significant differences between samples. Kolmogorov-Smirnov and Mann-Whitney U tests were used to compare two separate groups. p values are denoted through the text as follows: *p < 0.05; **p < 0.01; ***p < 0.001; ****p < 0.0001; p < 0.05 or lower values were considered significantly different when compared by one-way Kruskal-Wallis (Dunns’s posthoc test) or Kolmogorov-Smirnov and Mann-Whitney U tests. Thorough the text, the Box whisker plots represent median (line), mean (point), 25–75 percentile (box), 10–90 percentile (whisker), 1–99 percentile (X) and min - max (−) ranges.

## Supplementary information

Supplementary Information

## Data Availability

The data generated and analyzed during this study are available from the corresponding authors on reasonable request. We also show uncropped original blots in the Supplementary Material.

## References

[1] Apostolides PF, Trussell LO (2013). Rapid, activity-independent turnover of vesicular transmitter content at a mixed glycine/GABA synapse. J. Neurosci..

[2] Gomeza J (2003). Deletion of the Mouse Glycine Transporter 2 Results in a Hyperekplexia Phenotype and Postnatal Lethality. Neuron.

[3] Rousseau F, Aubrey KR, Supplisson S (2008). The glycine transporter GlyT2 controls the dynamics of synaptic vesicle refilling in inhibitory spinal cord neurons. J. Neurosci..

[4] Suhren O, Bruyn GW, Tuynman JA (1966). Hyperexplexia: A hereditary startle syndrome. J. Neurol. Sci..

[5] Harvey, R. J., Topf, M., Harvey, K. & Rees, M. I. *The genetics of hyperekplexia: more than startle! Trends in Genetics* 24, 439–447 (Elsevier Current Trends, 2008). 10.1016/j.tig.2008.06.005 18707791

[6] Carta E (2012). Mutations in the GlyT2 gene (SLC6A5) are a second major cause of startle disease. J. Biol. Chem..

[7] Lynch JW, Callister RJ (2006). Glycine receptors: a new therapeutic target in pain pathways. Curr. Opin. Investig. Drugs.

[8] Grothe B (2003). Sensory systems: New roles for synaptic inhibition in sound localization. Nat. Rev. Neurosci..

[9] Zafra F, Ibáñez I, Giménez C (2016). Glycinergic transmission: glycine transporter GlyT2 in neuronal pathologies. Neuronal Signal..

[10] Fornes A (2008). Trafficking properties and activity regulation of the neuronal glycine transporter GLYT2 by protein kinase C. Biochem J.

[11] de Juan-Sanz J, Zafra F, López-Corcuera B, Aragón C (2011). Endocytosis of the neuronal glycine transporter GLYT2: role of membrane rafts and protein kinase C-dependent ubiquitination. Traffic.

[12] Jiménez E (2011). P2Y purinergic regulation of the glycine neurotransmitter transporters. J. Biol. Chem..

[13] Villarejo-López L (2017). P2X receptors up-regulate the cell-surface expression of the neuronal glycine transporter GlyT2. Neuropharmacology.

[14] Armsen W, Himmel B, Betz H, Eulenburg V (2007). The C-terminal PDZ-ligand motif of the neuronal glycine transporter GlyT2 is required for efficient synaptic localization. Mol. Cell. Neurosci..

[15] Horiuchi M, Loebrich S, Brandstaetter JH, Kneussel M, Betz H (2005). Cellular localization and subcellular distribution of Unc-33-like protein 6, a brain-specific protein of the collapsin response mediator protein family that interacts with the neuronal glycine transporter 2. J. Neurochem..

[16] Geerlings A, Núñez E, López-Corcuera B, Aragón C (2001). Calcium- and syntaxin 1-mediated trafficking of the neuronal glycine transporter GLYT2. J. Biol. Chem..

[17] Arribas-González E, de Juan-Sanz J, Aragón C, López-Corcuera B (2015). Molecular basis of the dominant negative effect of a glycine transporter 2 mutation associated with hyperekplexia. J. Biol. Chem..

[18] Arribas-González E, Alonso-Torres P, Aragón C, López-Corcuera B (2013). Calnexin-assisted biogenesis of the neuronal glycine transporter 2 (GlyT2). PLoS One.

[19] de Juan-Sanz J (2013). Na+/K+-ATPase is a new interacting partner for the neuronal glycine transporter GlyT2 that downregulates its expression *in vitro* and *in vivo*. J. Neurosci..

[20] de Juan-Sanz, J. *et al*. Presynaptic control of glycine transporter 2 (GlyT2) by physical and functional association with plasma membrane Ca2+-ATPase (PMCA) and Na+-Ca2+ exchanger (NCX). *J. Biol. Chem*. M114.586966-, 10.1074/jbc.M114.586966 (2014). 10.1074/jbc.M114.586966 PMC4256361 25315779

[21] de Juan-Sanz J (2013). Constitutive endocytosis and turnover of the neuronal glycine transporter GlyT2 is dependent on ubiquitination of a C-terminal lysine cluster. PLoS One.

[22] DiAntonio A (2001). Ubiquitination-dependent mechanisms regulate synaptic growth and function. Nature.

[23] Pinto, M. J. *et al*. The proteasome controls presynaptic differentiation through modulation of an on-site pool of polyubiquitinated conjugates. *J. Cell Biol*. **212** (2016). 10.1083/jcb.201509039 PMC4810304 27022091

[24] Schwarz LA, Patrick GN (2012). Ubiquitin-dependent endocytosis, trafficking and turnover of neuronal membrane proteins. Mol. Cell. Neurosci..

[25] Berndsen CE, Wolberger C (2014). New insights into ubiquitin E3 ligase mechanism. Nat. Struct. Mol. Biol..

[26] Nakayama KI, Nakayama K (2006). Ubiquitin ligases: cell-cycle control and cancer. Nat. Rev. Cancer.

[27] Deshaies RJ, Joazeiro CAP (2009). RING Domain E3 Ubiquitin Ligases. Annu. Rev. Biochem..

[28] Rice DS, Northcutt GM, Kurschner C (2001). The Lnx family proteins function as molecular scaffolds for Numb family proteins. Mol. Cell. Neurosci..

[29] Weiss, A., Baumgartner, M., Radziwill, G., Dennler, J. & Moelling, K. c-Src is a PDZ interaction partner and substrate of the E3 ubiquitin ligase Ligand-of-Numb protein X1. FEBS Letters 581 (2007). 10.1016/j.febslet.2007.09.062 17936276

[30] Wolting CD (2011). Biochemical and Computational Analysis Of LNX1 Interacting Proteins. PLoS One.

[31] Higa S, Tokoro T, Inoue E, Kitajima I, Ohtsuka T (2007). The active zone protein CAST directly associates with Ligand-of-Numb protein X. Biochem. Biophys. Res. Commun..

[32] Lenihan, J. A. *et al*. Decreased Anxiety-Related Behaviour but Apparently Unperturbed NUMB Function in Ligand of NUMB Protein-X (LNX) 1/2 Double Knockout Mice. *Mol. Neurobiol*, 10.1007/s12035-016-0261-0 (2016). 10.1007/s12035-016-0261-0 27889896

[33] Young, P. W. LNX1/LNX2 proteins: functions in neuronal signaling and beyond. *Neuronal Signal* (2018). 10.1042/NS20170191 PMC7373230 32714586

[34] Liu X-D (2018). Retrograde regulation of mossy fiber axon targeting and terminal maturation via postsynaptic Lnx1. J. Cell Biol..

[35] Guo Z (2012). Proteomics strategy to identify substrates of LNX, a PDZ domain-containing E3 ubiquitin ligase. J. Proteome Res..

[36] Song E (2006). A high efficiency strategy for binding property characterization of peptide-binding domains. Mol. Cell. Proteomics.

[37] Lenihan JA, Saha O, Young PW (2017). Proteomic analysis reveals novel ligands and substrates for LNX1 E3 ubiquitin ligase. PLoS One.

[38] Xie P (2014). The covalent modifier Nedd8 is critical for the activation of Smurf1 ubiquitin ligase in tumorigenesis. Nat. Commun..

[39] Nguyen AT (2017). UBE2O remodels the proteome during terminal erythroid differentiation. Science.

[40] Hicke L (2001). Protein regulation by monoubiquitin. Nat. Rev. Mol. Cell Biol..

[41] Weissman AM (2001). Themes and variations on ubiquitylation. Nat. Rev. Mol. Cell Biol..

[42] Nie J (2002). LNX functions as a RING type E3 ubiquitin ligase that targets the cell fate determinant Numb for ubiquitin-dependent degradation. EMBO J..

[43] Young P (2005). LNX1 is a perisynaptic Schwann cell specific E3 ubiquitin ligase that interacts with ErbB2. Mol. Cell. Neurosci..

[44] Takahashi S (2009). The E3 ubiquitin ligase LNX1p80 promotes the removal of claudins from tight junctions in MDCK cells. J. Cell Sci..

[45] D’Agostino M (2011). Ligand of Numb proteins LNX1p80 and LNX2 interact with the human glycoprotein CD8 and promote its ubiquitylation and endocytosis. J. Cell Sci..

[46] Dho SE (1998). The mammalian numb phosphotyrosine-binding domain. Characterization of binding specificity and identification of a novel PDZ domain-containing numb binding protein, LNX. J. Biol. Chem..

[47] Bekri, A., Liao, M. & Drapeau, P. Glycine Regulates Neural Stem Cell Proliferation During Development via Lnx1-Dependent Notch Signaling. *Front. Mol. Neurosci*, 10.3389/fnmol.2019.00044 (2019). 10.3389/fnmol.2019.00044 PMC6387910 30833887

[48] Fioravante, D. *et al*. Protein kinase C is a calcium sensor for presynaptic short-term plasticity. *Elife* **3** (2014). 10.7554/eLife.03011 PMC5841930 25097249

[49] Shapira, R., Silberberg, S. D., Ginsburg, S. & Rahamimoff, R. Activation of protein kinase C augments evoked transmitter release. *Nature*, 10.1038/325058a0 (1987). 10.1038/325058a0 2432432

[50] Vaughan PF, Walker JH, Peers C (1998). The regulation of neurotransmitter secretion by protein kinase C. Mol. Neurobiol..

[51] Rees MI (2006). Mutations in the gene encoding GlyT2 (SLC6A5) define a presynaptic component of human startle disease. Nat. Genet..

[52] Flynn M, Saha O, Young P (2011). Molecular evolution of the LNX gene family. BMC Evol. Biol..

[53] Zheng N, Wang P, Jeffrey PD, Pavletich NP (2000). Structure of a c-Cbl-UbcH7 complex: RING domain function in ubiquitin-protein ligases. Cell.

[54] Lorick KL (1999). RING fingers mediate ubiquitin-conjugating enzyme (E2)-dependent ubiquitination. Proc. Natl. Acad. Sci. USA.

[55] Lenihan JA, Saha O, Mansfield LM, Young PW (2014). Tight, cell type-specific control of LNX expression in the nervous system, at the level of transcription, translation and protein stability. Gene.

[56] Liu Y, Beyer A, Aebersold R (2016). On the Dependency of Cellular Protein Levels on mRNA Abundance. Cell.

[57] Metzger MB, Pruneda JN, Klevit RE, Weissman AM (2014). RING-type E3 ligases: master manipulators of E2 ubiquitin-conjugating enzymes and ubiquitination. Biochim. Biophys. Acta.

[58] Rinetti GV, Schweizer FE (2010). Ubiquitination Acutely Regulates Presynaptic Neurotransmitter Release in Mammalian Neurons. J. Neurosci..

[59] Nishizaki, T. & Ikeuchi, Y. Activation of endogenous protein kinase C enhances currents through α1 and α2 glycine receptor channels. *Brain Res*, 10.1016/0006-8993(95)00543-Y (1995). 10.1016/0006-8993(95)00543-y 7583309

[60] Sato K, Betz H, Schloss P (1995). The recombinant GABA transporter GAT1 is downregulated upon activation of protein kinase C. FEBS Lett..

[61] Barrera, S. P. *et al*. PKC-dependent GlyT1 ubiquitination occurs independent of phosphorylation: Inespecificity in lysine selection for ubiquitination. *PLoS One*, 10.1371/journal.pone.0138897 (2015). 10.1371/journal.pone.0138897 PMC4587969 26418248

[62] Jayanthi LD, Samuvel DJ, Blakely RD, Ramamoorthy S (2005). Evidence for biphasic effects of protein kinase C on serotonin transporter function, endocytosis, and phosphorylation. Mol. Pharmacol..

[63] Qian Y (1997). Protein kinase C activation regulates human serotonin transporters in HEK-293 cells via altered cell surface expression. J. Neurosci..

[64] Sorkina T (2006). RNA interference screen reveals an essential role of Nedd4-2 in dopamine transporter ubiquitination and endocytosis. J. Neurosci..

[65] Hong WC, Amara SG (2013). Differential targeting of the dopamine transporter to recycling or degradative pathways during amphetamine- or PKC-regulated endocytosis in dopamine neurons. FASEB J..

[66] González-González, I. M., García-Tardón, N., Giménez, C. & Zafra, F. PKC-dependent endocytosis of the GLT1 glutamate transporter depends on ubiquitylation of lysines located in a C-terminal cluster. *Glia*, 10.1002/glia.20670 (2008). 10.1002/glia.20670 18381652

[67] Zafra F (1995). Glycine transporters are differentially expressed among CNS cells. J. Neurosci..

[68] Núñez E (2009). Subcellular localization of the neuronal glycine transporter GLYT2 in brainstem. Traffic.

