# Supplementary material for: E3 ubiquitin ligases LNX1 and LNX2 are major regulators of the presynaptic glycine transporter GlyT2
Source: Sci Rep. 2019 Oct 18;9:14944. doi: 10.1038/s41598-019-51301-x (PMC6802383; doi:10.1038/s41598-019-51301-x)

E3 ubiquitin ligases LNX1 and LNX2 are major regulators of the presynaptic glycine transporter GlyT2

de la Rocha-Muñoz A[+1,2], Núñez E[+1,2], Arribas-González E[+3], López-Corcuera B[1,2], Aragón C[*1,2] & de Juan-Sanz J[*4]

Supplementary Information

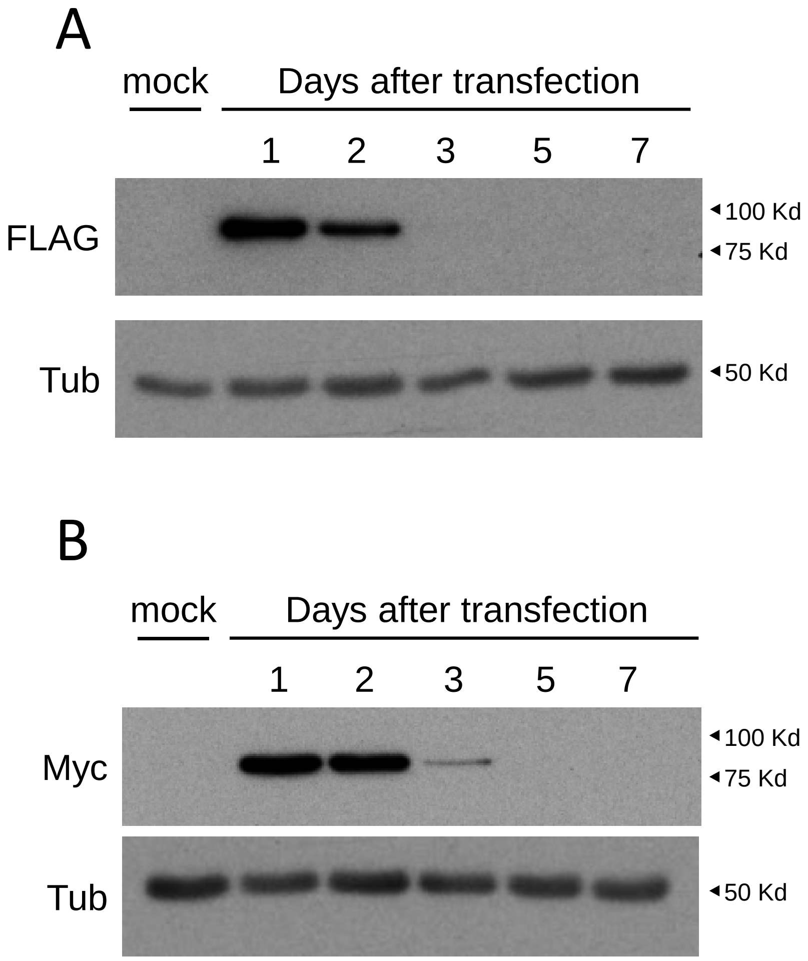

Supplementary 1. LNX proteins present a very short half-life. Cortical neurons expressing FLAG-p80LNX1 (A) or Myc-LNX2 (B) were lysed. Immunodetection against FLAG, Myc and tubulin was performed.

Uncropped blots

Uncropped blots Fig 2A

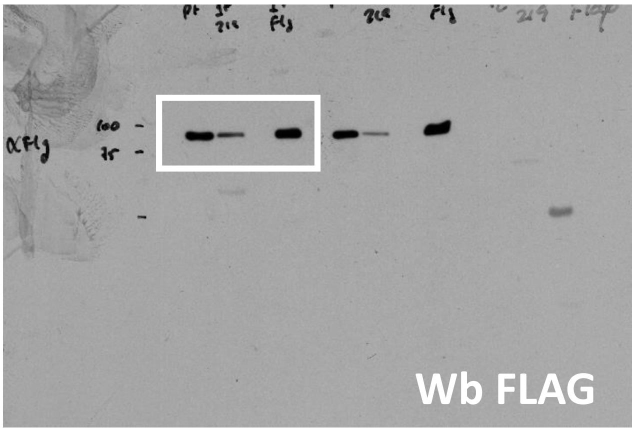

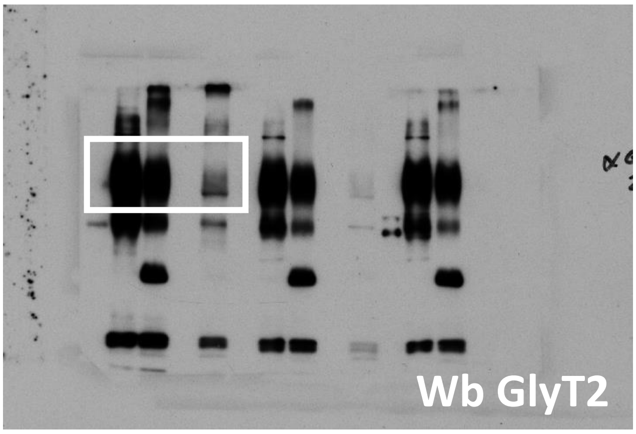

Uncropped blots Fig 2B

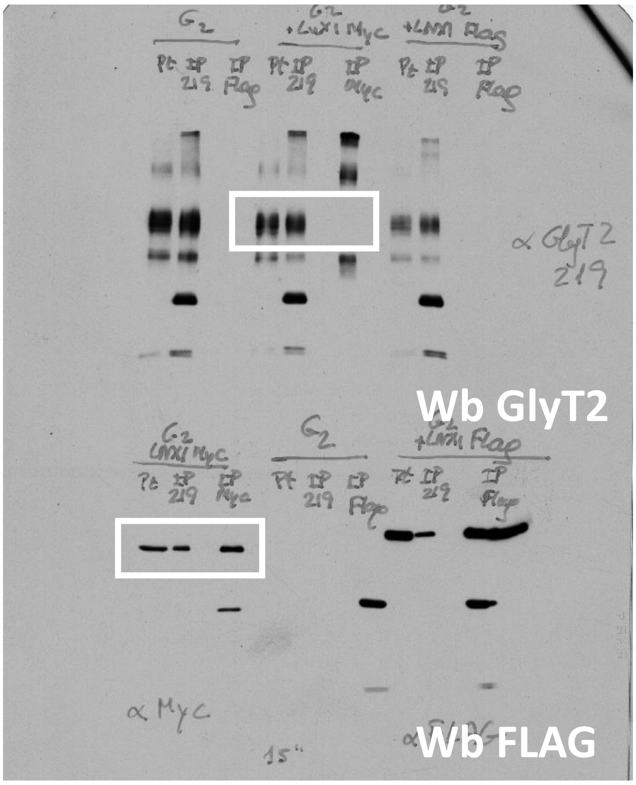

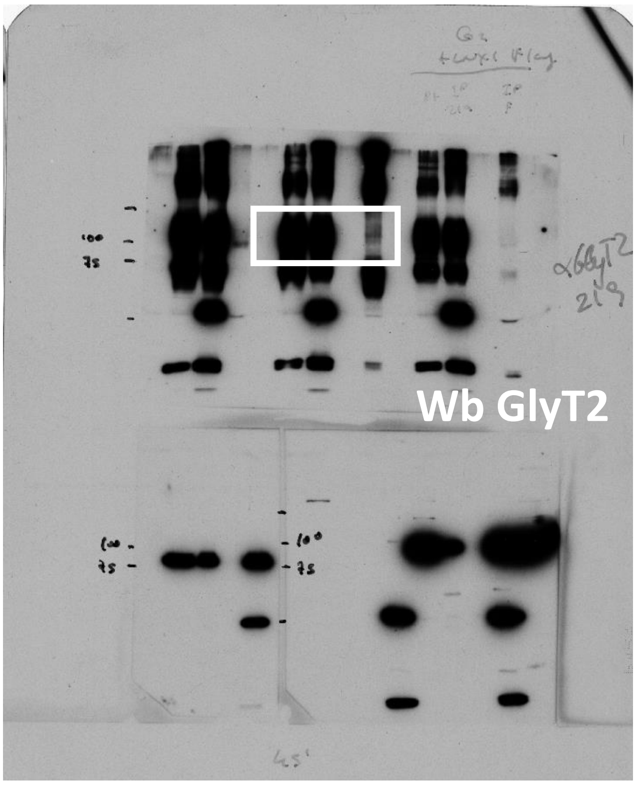

Uncropped blots Fig 2C: GlyT2

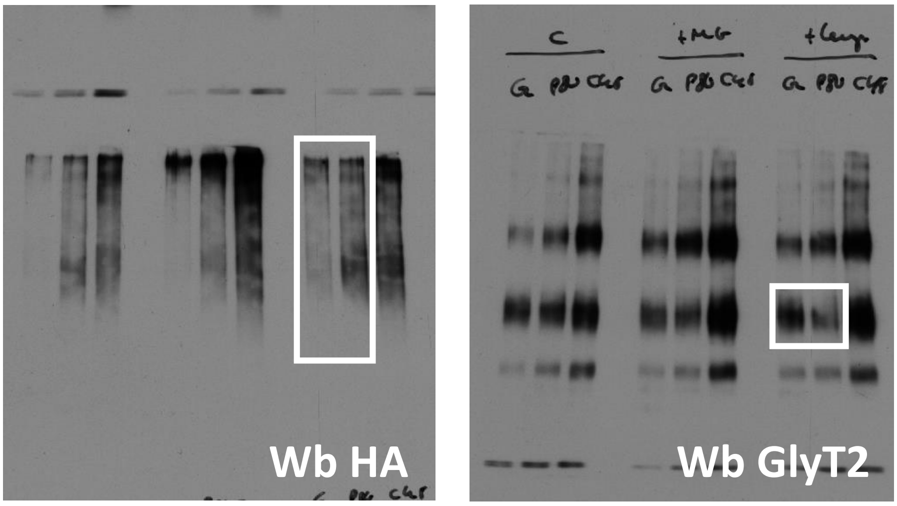

Uncropped blots Fig 2C: GlyT2-4KR

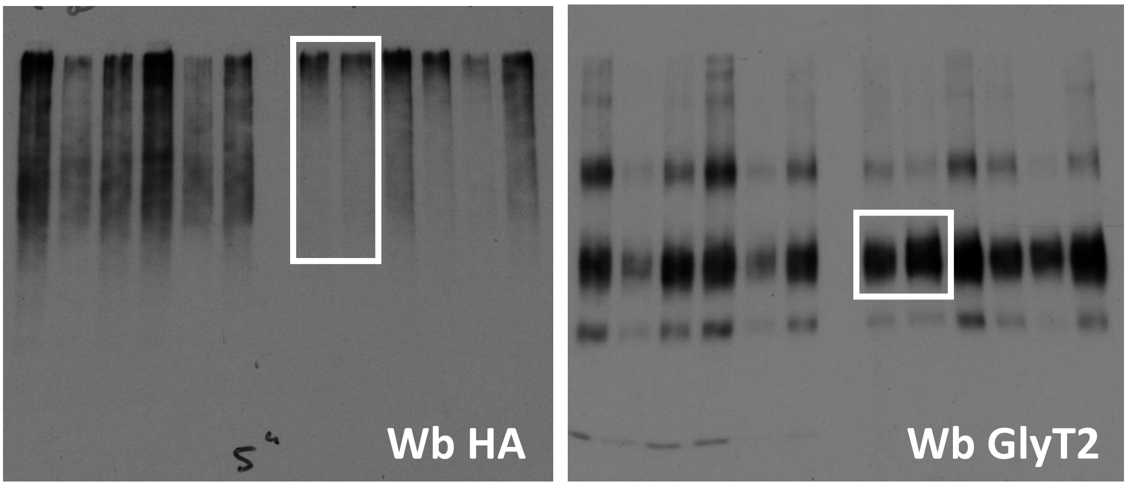

Uncropped blots Fig 2E: GlyT2

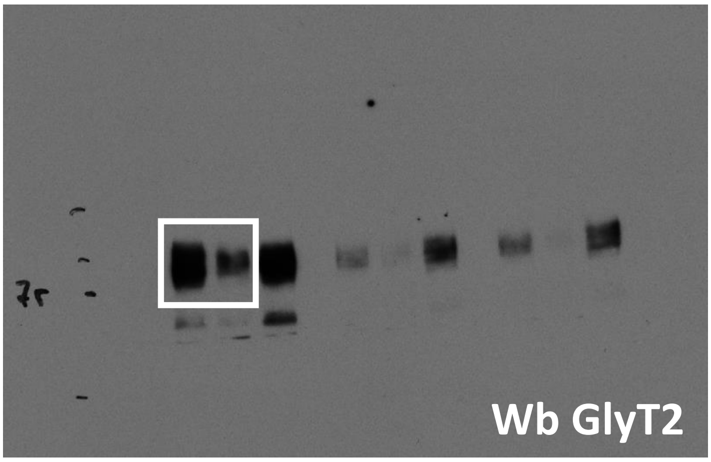

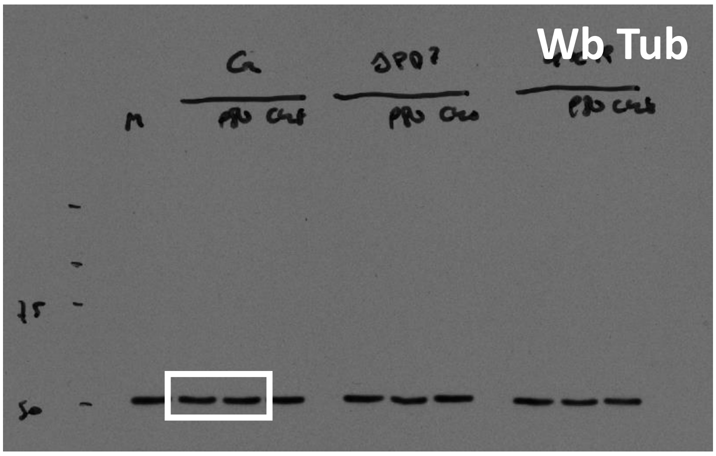

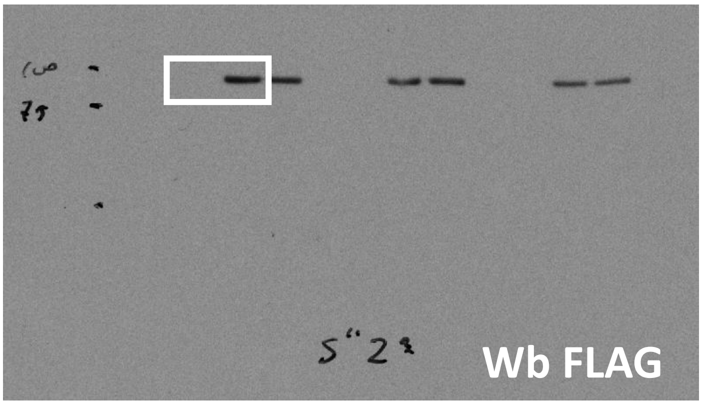

Uncropped blots Fig 2E: GlyT2-4KR

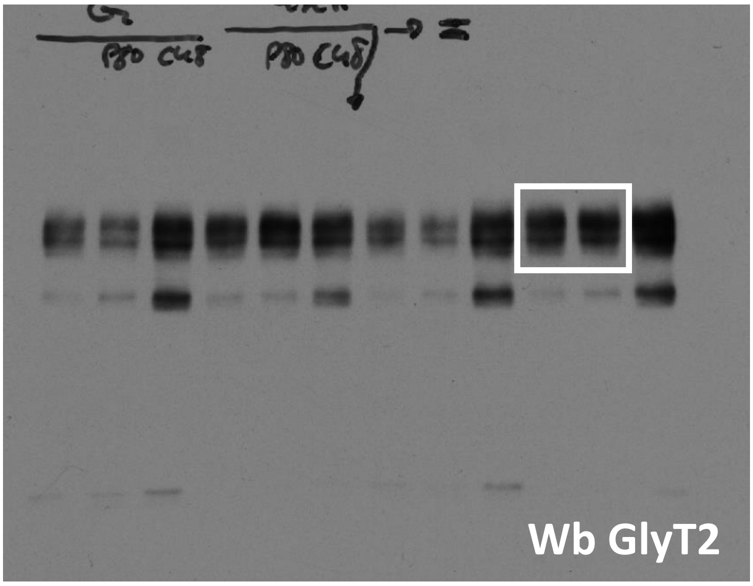

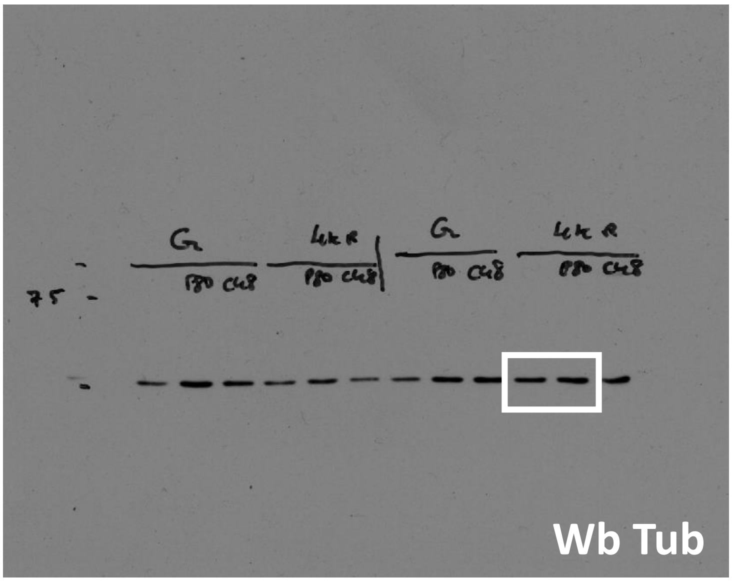

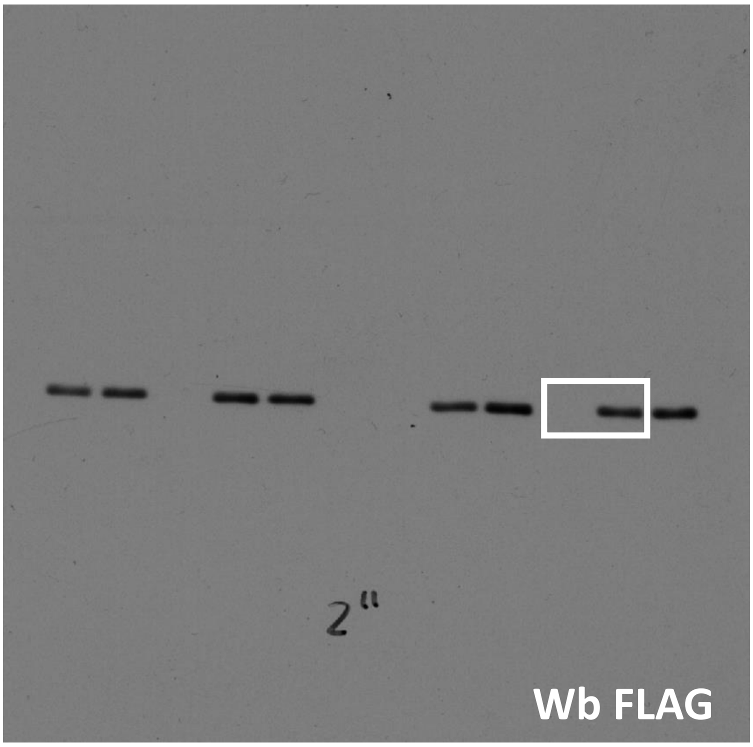

Uncropped blots Fig 3A

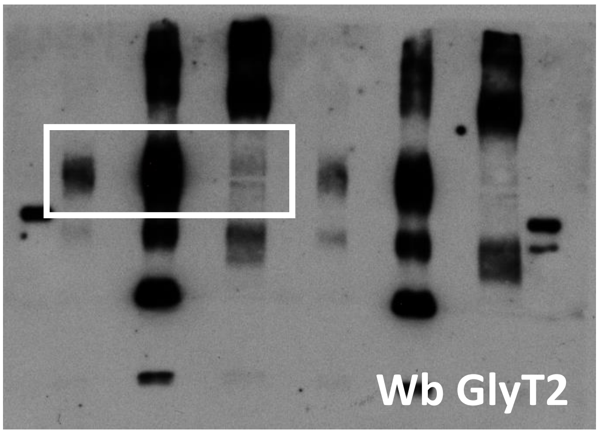

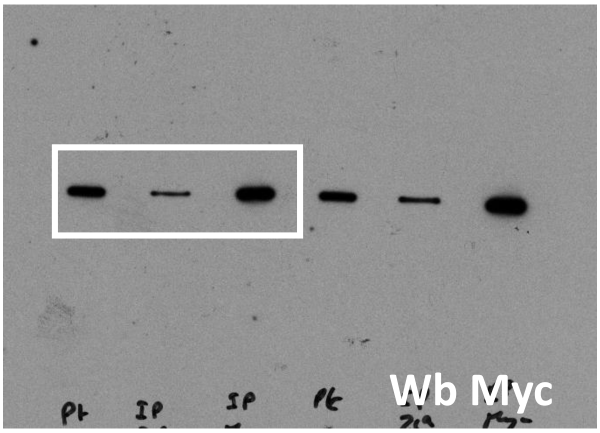

Uncropped blots Fig 3B

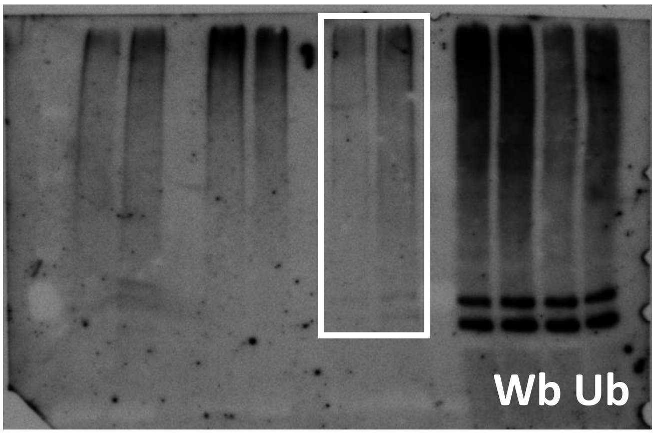

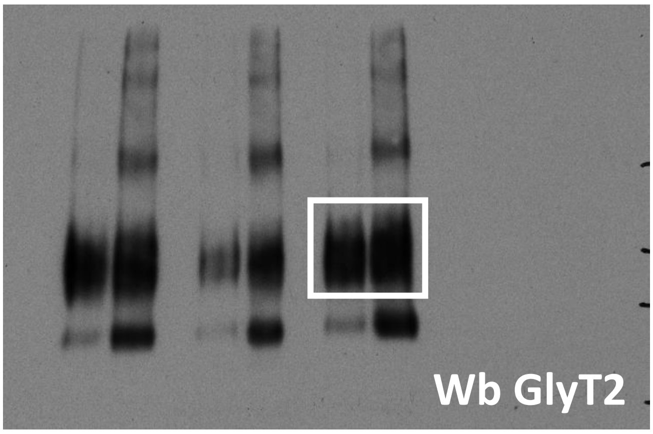

Uncropped blots Fig 3D:GlyT2

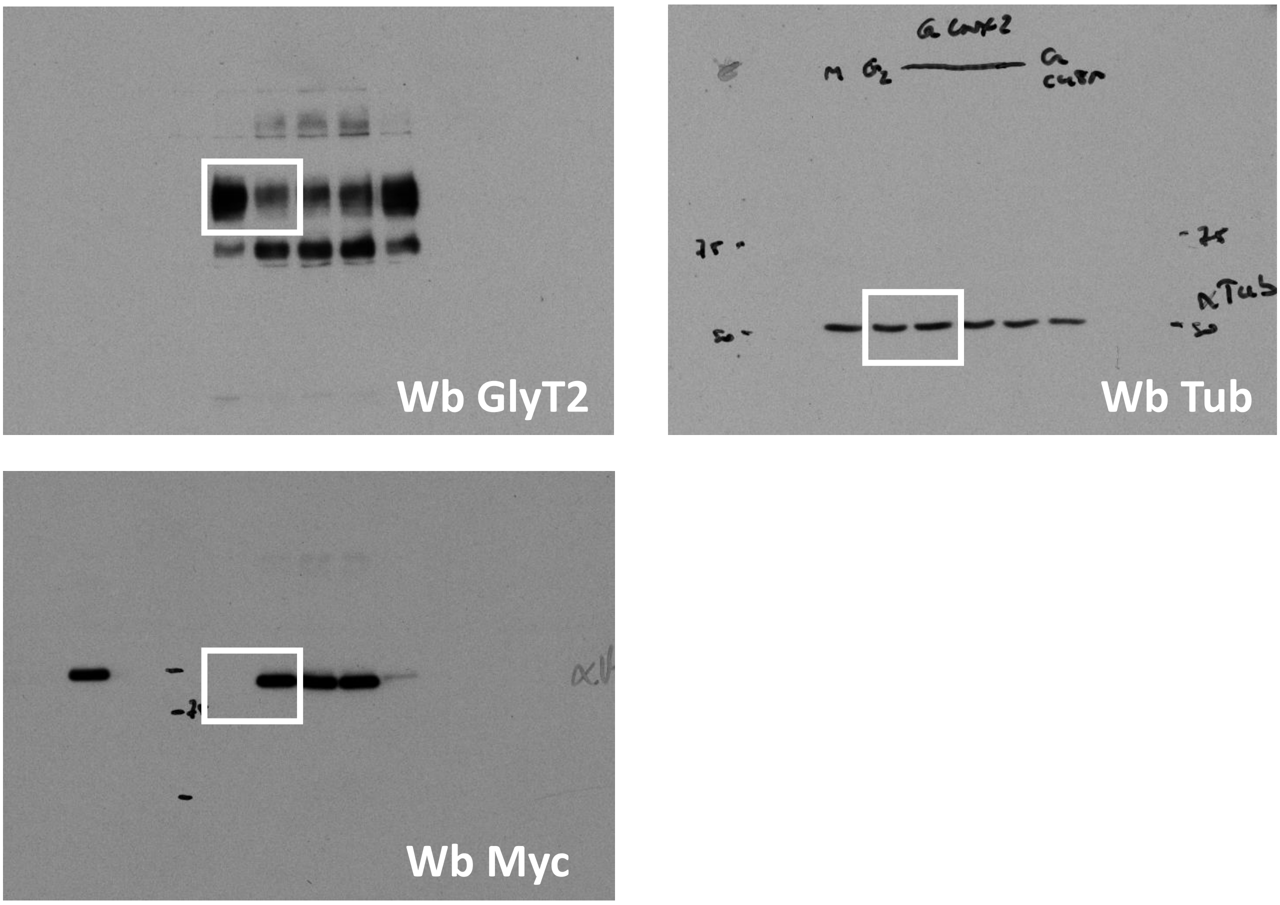

Uncropped blots Fig 3D:GlyT2-4KR

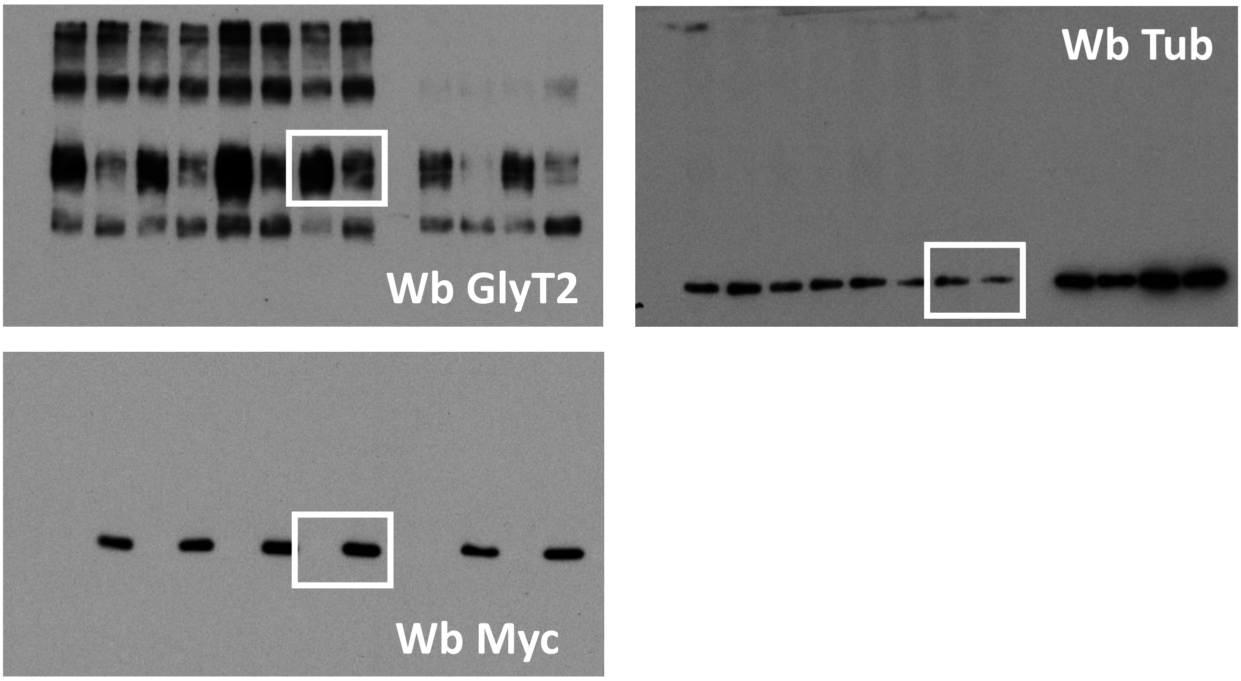

Uncropped blots Fig 4C

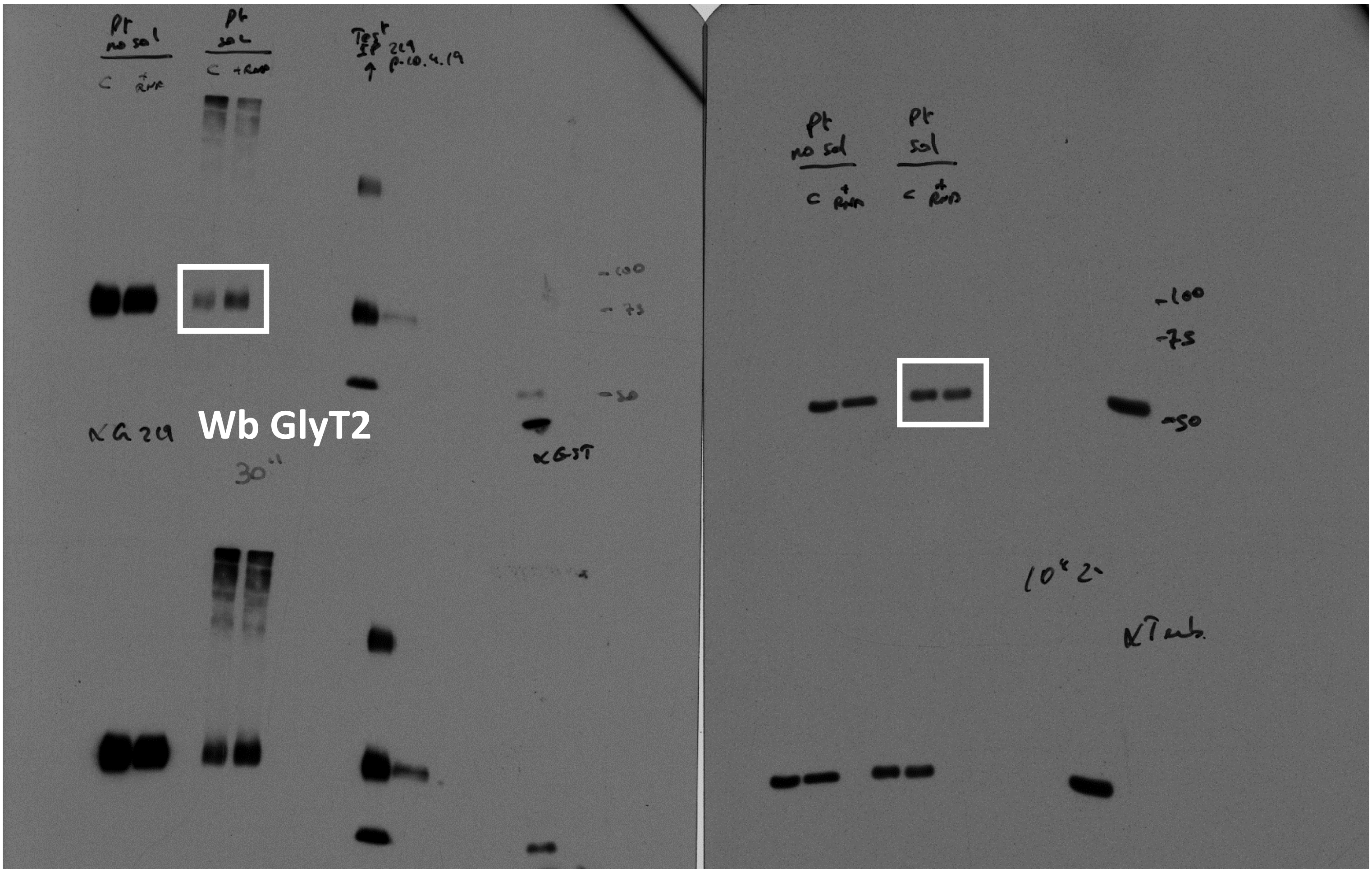

Uncropped blots Fig 4E

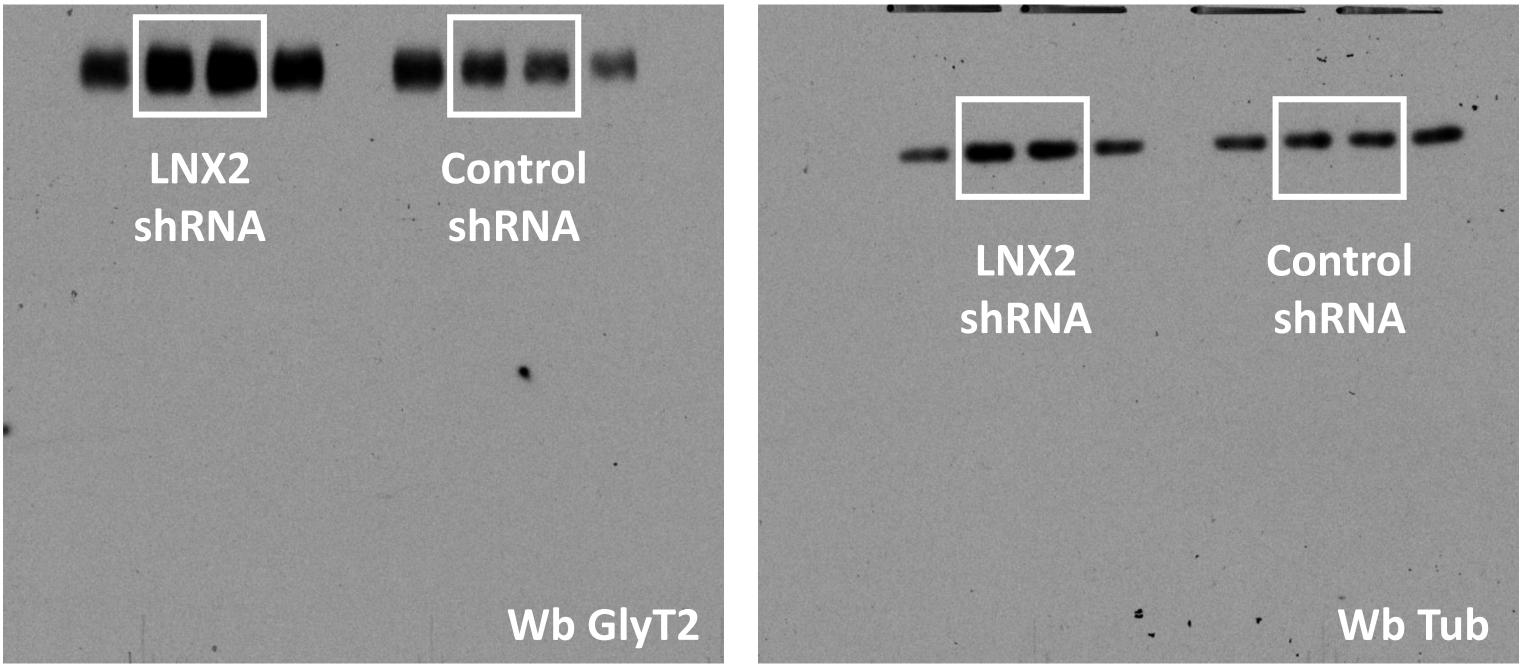

Supplement: Supplementary file 1 — Supplementary Information [file 41598_2019_51301_MOESM1_ESM.pdf]
